# Supplementary material for: Time to sputum culture conversion, treatment outcomes, and associated factors among rifampicin-resistant or multidrug-resistant tuberculosis patients in the Sidama region, Ethiopia: A retrospective follow-up study
Source: PLoS One. 2026 Apr 13;21(4):e0327705. doi: 10.1371/journal.pone.0327705 (PMC13075698; doi:10.1371/journal.pone.0327705)
Supplement: S1 File — (PDF) [file pone.0327705.s001.pdf]

S1_Form. Data Collection form for time to sputum culture conversion treatment outcome and the associate factors among rifampicin-resistant or multidrug-resistant tuberculosis patients in the Sidama region Ethiopia

Instructions: Complete this form by extracting data from the patient's medical record and MDR-TB treatment register. Refer to the patient's MRN and MDRN for verification.

Section 1: Patient Identification

Field	Response
ID	__________
MRN	__________
MDRN	__________

Section 2: Demographic Information

Field	Response
Sex	☐ Male ☐ Female
Age (years)	__________

Section 3: Clinical Characteristics

Field	Response
HIV status	☐ HIV-positive ☐ HIV-negative
Resistance type	☐ RR-TB ☐ MDR-TB
Patient registration group	☐ New ☐ Relapse ☐ Loss to follow-up ☐ Treatment failure
Treatment regimen	☐ Short-term ☐ Long term

Section 4: Treatment Outcome

Outcome	Yes	No
Cured	☐	☐
Treatment completed	☐	☐
Died	☐	☐
Treatment failure	☐	☐
Loss to follow-up	☐	☐
Not evaluated	☐	☐

Section 5: Sputum Culture Conversion

Time Point	Date	Result
Baseline culture	________	☐ Positive ☐ Negative
Month 1	___________	☐ Positive ☐ Negative
Month 2	___________	☐ Positive ☐ Negative
Month 3	___________	☐ Positive ☐ Negative
Month 4	___________	☐ Positive ☐ Negative
Month 6	___________	☐ Positive ☐ Negative
Month 8	___________	☐ Positive ☐ Negative
Month 12	___________	☐ Positive ☐ Negative

Section 6: Sputum Culture Reversion

Field	Response
Culture reversion	☐ Yes If yes, date of reversion ___________ ☐ No

Definition: Culture reversion is the time between the date of the second negative culture marking culture conversion and the first of the two positive cultures that indicate reversion
