# Supplementary material for: Time to sputum culture conversion, treatment outcomes, and associated factors among rifampicin-resistant or multidrug-resistant tuberculosis patients in the Sidama region, Ethiopia: A retrospective follow-up study
Source: PLoS One. 2026 Apr 13;21(4):e0327705. doi: 10.1371/journal.pone.0327705 (PMC13075698; doi:10.1371/journal.pone.0327705)
Supplement: Attachment — Submitted filename: Response to Reviewers.docx [file pone.0327705.s002.docx]

**To**
The Editor and Reviewers
PLOS ONE

**Date:** October 30, 2025

**Subject:** Response to Reviewer Comments for Manuscript ID: PONE-D-25-32357

Dear Editor and Reviewers,

We sincerely thank you for your valuable feedback, which has greatly contributed to improving the quality and clarity of our work. In response to your insightful comments, we have carefully revised the manuscript and addressed each concern to the best of our ability.

For ease of review, we have provided a detailed, point-by-point response below, indicating how each comment has been addressed. The corresponding revisions in the manuscript are highlighted in yellow for clarity.

We deeply appreciate your time and constructive engagement, and we kindly request your continued support as our manuscript undergoes further evaluation. Should you have any additional suggestions, we would be grateful for your guidance in further strengthening our work.

Thank you once again for your thoughtful review and consideration.

Sincerely,
Wolde Abreham Geda
On behalf of all authors

The Editor’s comments

- A rebuttal letter that responds to each point raised by the academic editor and reviewers was uploaded to the separate file labeled 'Response to Reviewers'.
- A marked-up copy of the manuscript that highlights changes made to the original version was uploaded to the separate file labeled 'Revised Manuscript with Track Changes'.
- An unmarked version of the revised paper without tracked changes was uploaded to the separate file labeled 'Manuscript'

**Journal requirements**

1. Please ensure that your manuscript meets PLOS ONE's style requirements, including those for file naming.

We confirm that the manuscript has been formatted in accordance with the PLOS ONE style requirements. The file naming and formatting have been completed using the official PLOS ONE style templates available at <https://journals.plos.org/plosone/s/file?id=wjVg/PLOSOne_formatting_sample_main_body> and <https://journals.plos.org/plosone/s/file?id=ba62/PLOSOne_formatting_sample_title_authors_affiliations.pdf>.

2. If the reviewer comments include a recommendation to cite specific previously published works, please review and evaluate these publications to determine whether they are relevant and should be cited. There is no requirement to cite these works unless the editor has indicated otherwise.

The reviewers did not recommend citing any specific previously published works, and the editor has not indicated the need to include additional citations. Therefore, no changes were required in this regard.

**Responses to the reviewers’ comments**

1. Is the manuscript technically sounds, and do the data support the conclusions?
The manuscript must describe a technically sound piece of scientific research with data that supports the conclusions. Experiments must have been conducted rigorously, with appropriate controls, replication, and sample sizes. The conclusions must be drawn appropriately based on the data presented.

Reviewer #1: Partly

Reviewer #2: No

We appreciate the reviewers’ comments regarding the rigor and data validity of our study. As this was a retrospective single-arm cohort study, involving patients with MDR/RR-TB, establishing an unexposed control group was not feasible due to ethical and practical constraints. Instead, we ensured methodological rigor through consistent data collection procedures, inclusion of all eligible patients within the study period, and appropriate statistical analyses to strengthen the internal validity of our findings. We have also reviewed the entire manuscript to confirm that the data presented accurately support the conclusions drawn.

2. Has the statistical analysis been performed appropriately and rigorously?

Reviewer #1: No

Reviewer #2: I Don't Know

We appreciate the reviewers’ feedback regarding the statistical analysis. In response, we have thoroughly re-evaluated and re-run all statistical analyses to ensure rigor and appropriateness. Specifically, we have:

Declared the data as survival data and applied appropriate survival analysis methods;

Checked and verified the proportional hazards assumption;

Conducted model selection based on statistical criteria;

Assessed model fit to confirm adequacy; and carefully reviewed data entry, analysis procedures, and interpretation of results.

These steps have been reflected in the revised manuscript to ensure transparency and methodological soundness.

3. Have the authors made all data underlying the findings in their manuscript fully available?

The [PLOS Data policy](http://track.editorialmanager.com/CL0/http:%2F%2Fwww.plosone.org%2Fstatic%2Fpolicies.action%23sharing/1/010f019950a6493b-af0d59ad-c452-41ff-8adb-d387bbde7e11-000000/qU_W1X4ZQKlvkMAJJKQd4AHx2gXwzx_jOq5k2M7ebiA=228) requires authors to make all data underlying the findings described in their manuscript fully available without restriction, with rare exception (please refer to the Data Availability Statement in the manuscript PDF file). The data should be provided as part of the manuscript or its supporting information, or deposited to a public repository. For example, in addition to summary statistics, the data points behind means, medians and variance measures should be available. If there are restrictions on publicly sharing data—e.g. participant privacy or use of data from a third party—those must be specified.

Reviewer #1: Yes

Reviewer #2: No

All data associated with this study were made available at the time of the initial submission. The complete manuscript is posted as a preprint on medRxiv. We would be pleased to furnish any further information or materials upon request.

4. Is the manuscript presented in an intelligible fashion and written in Standard English?

PLOS ONE does not copyedit accepted manuscripts, so the language in submitted articles must be clear, correct, and unambiguous. Any typographical or grammatical errors should be corrected at revision, so please note any specific errors here.

Reviewer #1: No

Reviewer #2: No

We tried to clear any typographical and grammatical errors in our revised manuscript to the best of our knowledge.

5. Review Comments to the Author

Please use the space provided to explain your answers to the questions above. You may also include additional comments for the author, including concerns about dual publication, research ethics, or publication ethics. (Please upload your review as an attachment if it exceeds 20,000 characters)

Reviewer #1: Critical comments

1- Review the sample size calculation and sampling strategy. The description is confusing. The authors calculated a sample size and designed a sampling plan, but in the end included all patients without reaching the calculated N. The authors must clearly state what was actually done, i.e., the inclusion of all eligible individuals, and, if appropriate, calculate the statistical power of the achieved sample based on the observed differences.
We appreciate the reviewer’s valuable comment. We have revised the section to clarify our sampling approach. Although a sample size was initially calculated, in practice all eligible individuals in the region were included in the study. This clarification has been incorporated into the Methods section of the revised manuscript.
2- Even though this is a retrospective study, patients were followed in some manner. The authors must specify how follow-up was conducted and for how long.

In response to the reviewer’s comment, we have clarified the follow-up process and its duration in the manuscript. The researchers conducted a retrospective follow-up of the study participants for a total of 29,001 person-days. We obtained follow-up information from the patients’ medical records, which included routine clinical visits and laboratory evaluations throughout the treatment period. The data were collected using a standardized form and encompassed demographic information (sex and age), clinical data (time to sputum culture conversion, treatment outcomes, and treatment regimens), as well as associated factors (HIV status and previous treatment history). All data entered into Microsoft Excel and subsequently imported into SPSS version 26 for analysis.

.3- Provide detailed definitions of all evaluated outcomes.

Definition of the outcome variables

Time-to-sputum culture conversion: the duration between the time of treatment initiation and the date of the first of the two consequent culture conversions, collected at least 30 days apart.

Time to culture conversion refers to the time required for an event to occur in this study.

Cured: a confirmed pulmonary TB case at the time of treatment initiation, who completed the treatment recommended by the national guideline, with evidence of bacteriological response and without evidence of failure.

Treatment completed: a patient who completed treatment recommended by the national policy, but the outcome does not fulfill the criteria of cure or treatment failure.

Treatment failed: a pulmonary TB patient whose treatment regimen needed to be stopped or changed permanently.

Died: a TB patient who died before the start of treatment or during the treatment course.

Lost to follow-up: a TB patient who did not start treatment or interrupted treatment for two consecutive months or more.

Not evaluated: a TB patient with no treatment outcome judgment.

Treatment success: is defined as the total number of patients who were cured and those who completed treatment

Culture reversion: time to culture reversion is the time between the date of the second negative culture marking culture conversion and the first of the two positive cultures that indicate reversion.

4- The study included multiple treatment records with different timeframes, resulting in unbalanced populations across treatment groups. The authors need to explain how they addressed this source of bias.

We thank the reviewer for insightful comments, regarding the potential bias arising from unbalanced populations across treatment groups. Of course, our study aimed at comparing the effectiveness of the treatment regimens in terms of time to culture conversion and treatment outcomes. However, we observed no significant differences in treatment outcomes among the groups, while we acknowledge that unequal sample sizes could introduce bias. We included all eligible participants to ensure a comprehensive analysis. The participants were not left out to artificially balance the groups. The revised manuscript has explicitly discussed the inherent imbalance as a limitation in the section.

5- The inclusion of relapse cases introduces an established risk factor for treatment abandonment. The authors must clarify how they accounted for this in the analysis.

We conducted a statistical analysis to explore the potential association between explanatory variables, including patient registration groups, such as relapse and treatment abandonment, as per the reviewer's comment.

Factors associated with treatment abandonment among multidrug-resistant tuberculosis patients in the bivariable analysis, with a 95% CI, COR 3.2, 1-9.2, P = 0.026, included a history of previous treatment failure. However, this association was not significant in the multivariable analysis. In addition, there was no significant association between the current treatment abandonment and explanatory variables, such as sex, age, HIV status, drug resistance type, patient registration groups, and MDR-TB treatment regimens.

6- Provide a clear and detailed description of the statistical analyses. Specify which measures were calculated, which tests were used and for which variables, how assumptions were assessed, and how results were interpreted.

In response to the reviewer’s comments, we have revised the statistical analysis section of our manuscript. As a result, we gave the measures calculated, like frequencies, mean, median, and standard deviation.

We also outlined tests used to assess how the independent variables (sex, age, HIV infection, patient registration group, type of drug resistance, and treatment regimens) were associated with the outcome variables, time-to-sputum culture conversion, and treatment outcomes. We employed Akaike information criteria (AIC) for model selection. The model that has the lowest AIC was selected as the most efficient model. The Schoenfeld test is used to assess the fulfillment of the proportional hazard assumption. When the data were distributed around a horizontal line, it was considered that the data did not violate the proportional hazard assumption. In addition, the Cox-Snell residual tests are employed to assess the model fit to the data. When the residuals were roughly distributed around the diagonal line (X=Y, 45 degrees), the model was fit. The results with 95%CI, p value less than 0.05 were considered statistically significant.
7- Present a more detailed description of the Cox regression models. The authors must specify dependent and independent variables, adjustments performed how these adjustments were evaluated, and how results were reported.

After reviewing the reviewer’s comments, we revised our survival analysis model to identify the model that best fits our data and satisfies the proportional hazards assumption. Previously, we conducted our analysis using the Cox proportional hazards model due to its convenience and simplicity. However, upon evaluating the models using the Akaike Information Criterion (AIC), we found that the Weibull frailty gamma model (AIC = 600.7) provided the best fit for our dataset. Therefore, we have included a detailed description of the Weibull frailty gamma model, rather than the Cox regression model.

8- Revise the discussion to synthesize the main findings and critically interpret them in light of the study’s focus. The reported reduction in culture conversion time compared with other studies requires explanation. Could bias have contributed to this finding? What are the clinical and practical implications?

The time to sputum culture conversion observed in this study fell within the WHO-recommended period of 120 days for a favorable treatment outcome. The current finding suggests both effective treatment and good patient adherence in our study population. Furthermore, the inclusion of all eligible individuals minimized the potential for selection bias.

Despite a notably high rate of loss to follow-up, the majority of patients who remained in the study achieved culture conversion within an optimal timeframe. Moreover, we have explained the clinical and practical implications of these findings in the revised manuscript.

9- Discuss the implications of the treatment outcome findings for the management of patients with resistant or multidrug-resistant tuberculosis.

The higher rate of unfavorable treatment outcome, especially loss to follow-up and treatment failure requires substantial action to improve patient adherence. The implications were explained in the revised document.

10- Explicitly state how the described limitations affect the interpretation of results and indicate how the authors attempted to mitigate them.
We revised the limitation section and addressed the reviewer’s comment.
11- Clearly and consistently state the study objective. As currently written, the objective remains ambiguous.

We have revised according to the reviewer’s comment.

Major comments

1- Revise the “source and study population” section and the inclusion/exclusion criteria. These subsections are redundant.

We have revised the section according to the reviewer’s comments.
2- Reconsider the exclusion of individuals under 15 years of age. It is redundant to exclude a population that was not initially included in the study.

We removed the redundant individuals under the age of fifteen exclusion criteria.

3- Revise the ethics and informed consent subsections. These are redundant.

We revised the ethics and informed consent section to address the reviewer’s comment.

4- Strengthen the description of the study population. The current description is weak and insufficient to understand the characteristics of the participants.

We revised the section according to the comment.

5- Figure 2 requires better presentation in the Results section. The authors should specify which treatments were associated with shorter culture conversion times.
Revision made, and there was no any treatment which associated with time to culture conversion.
6- Revise the Conclusion. It should directly answer the research question and restate the study objective. Additional sentences belong in the Discussion section.
Revised according to the reviewer’s comment.
Minor comments

1- Figures require more detailed data presentation to facilitate interpretation. Improve figure quality.
We have provided more detailed information in the text report while keeping the figures more concise to avoid redundancy.
2- Revise the manuscript thoroughly to improve readability. Issues of textual cohesion and sentence connection reduce fluency.

We revised the manuscript in depth as per the reviewer’s comment.

**Reviewer #2: PLOS ONE**
Time-to-sputum culture conversion, treatment outcomes, and associated factors among multidrug-resistant tuberculosis patients in the Sidama region, Ethiopia: A retrospective follow-up study

The authors have conducted a retrospective follow-up study on 346 patients aimed to determine the time-to-culture conversion, treatment outcomes, and associated factors among multidrug-resistant tuberculosis in the Sidama Region, Ethiopia from 2013-2024.
The authors concluded that most of the patients (87.3%) achieved culture conversion within three months. The patients with a history of retreatment faced worse outcome, pointing to serious treatment follow-up to the patients.
Such studies are very important to evaluate the treatment and factors associated with failure to improve outcomes of patients with MDR-TB. Still, the manuscript seems preliminary and has several limitations and should be substantially revised to make their data more accessible for the reader.
We appreciate the reviewer’s insightful comments. As a result, we have undertaken substantial revisions throughout the entire manuscript.
The Introduction is partly repetitive on the importance of the study. Still, there is no background or references on what is already known and previously published about time-to-culture conversion, treatment outcomes, and associated factors (only in Discussion). Also, the 6 months BPaLm regimen is not mentioned (Line 72: “MDR-TB treatment takes longer, 18-20 months, and it is very costly”).
The document is revised according to the reviewer’s comment.

In the Methods the definitions of various parameters should be better explained. Ex. Line 171: short-term, long-term and standardized treatment should be explained since this could have relevance for side effects and outcome. I am not qualified to evaluate all the statistical methods used but the data collection and statistical method are not very well explained. Also, the implications of the sample size calculations are not clear as the authors did not use these data as the numbers of patients available were much fewer.

The treatment regimens have been explained in greater detail to enhance clarity and comprehensiveness in the revised manuscript, and as follows.

Long-term regimens are an extended treatment course for MDR/RR-TB. The treatment regimen typically lasts 18 months or longer. Depending on the patient’s drug resistance profile, previous treatment history, and tolerance to medications, the regimen can follow either a standardized or an individualized approach.

The standardized treatment: In this approach, all eligible patients receive uniform treatment.

The individualized treatment: in contrast, the eligible patients get treatment in a tailored manner, based on their drug susceptibility testing profile.

Short-term regimens are a novel, shorter standardized MDR/TB treatment approach, which extends from nine to twelve months.

The BPaLM regimen: this group of regimen is a 6-month all-oral treatment for MDR/RR-TB. It consists of Bedaquiline (B), Pretomanid (Pa), Linezolid (L), and Moxifloxacin (M).

The statistical methods section has been thoroughly revised to ensure accuracy and transparency.

The section on sample size determination has been revised to indicate that all eligible individuals were included in the study, thereby addressing both reviewers’ comments.

The discussion is partly repetitive of results and just state where their findings were similar or different from other studies without discussing or giving possible explanations for their findings. The Conclusion is also a repetition from Results and Discussion and Limitations were deficient.

We revised the section and gave the possible explanations.

The manuscript also needs substantial update and general editing concerning language, fonts (Tables), typing errors including correct and not repetitive use of abrev. as ex. Tuberculosis (TB) and Multidrug-resistant/ Rifampicin mono-resistant- TB (MDR/TB) that have been used several times.

The manuscript was reviewed according to the reviewer’s comment.

Line 34 needs ref. The WHO 2024 report will most likely provide data for 2023 including for Ethiopia.

We appreciate the reviewer’s observation and we revised it according to the reviewer’s comment. But, in the WHO 2024 report, some data were reported even from 2021 or 2021 reports.

Line 102: such a detailed description of the country is probably not needed.

The section was revised, according to the reviewer’s comment.

Line 121: It is not clear concerning the time period when the patient data were collected: “ The patients were registered from January 1, 2013, to June 30, 2024, from the Sidama region of Ethiopia at the Yirgalem treatment initiation center. The researchers accessed the patient data anonymously, using their medical and multidrug-resistant TB record number, from April 1 to December 31, 2024”.

We cleared the issue according to the comment.
Line 134: “…. patients with culture-negative results at baseline, and patients diagnosed with MDR/RR-TB who did not begin anti-TB treatment.” An explanation on who these patients are and why not treated would be interesting.

Some patients diagnosed with MDR/RR-TB using molecular methods, such as GeneXpert or line probe assay, may have negative culture results at baseline.. We excluded these patients because our study focused on the time to sputum culture conversion—from positive to negative—and therefore required culture-positive cases at baseline.

Additionally, a few patients who were culture positive did not initiate anti-TB treatment. It could be due to reasons such as treatment refusal, death before treatment initiation, or transfer out. Since our study objective was to assess culture conversion among treated patients, we excluded these individuals without investigating the specific reasons for non-initiation of therapy.

Line 142: not clear if the same calculations were performed for all outcomes? Also, since the largest sample size for a single-arm cohort study was = 1977, but the total eligible RR/MDR-TB population in the region during the study period was finally 346, are the study powered to answer the research questions and are the sample size calculations of any value?

We revised the sample size to include all eligible individuals. Although the total number of cases was smaller than the initially calculated sample size, including all available patients from this finite population enhances representativeness and provides the best possible power within the study setting.

Line 205: Participant agreement is already stated in the section above.

It was revised according to the reviewers’ comment.

Line 209: Write more precise concerning which patients included “A total of 420 patients with Multidrug-resistant tuberculosis enrolled over an 11-year follow-up”. This statement disagrees with Table 3 were also RR and INH resistance are included.

We rewrote the section according to the comment.
Line 262: People who registered with a drug-resistance type of MDR-TB were associated with early time to-sputum culture conversion with aHR 4.7 (1-21), P = 0.041 compared with the patients with a resistance type of RR-TB, while a treatment regimen of long-term MDR-TB treatment was significantly associated with an aHR of 10.1 (2.2-45), P = 0.003 compared with the short-term treatment. This statement is not so clear formulated and not as expected that MDR-TB should do better than rifampicin mono resistance? Still, the authors just state this and provides no possible explanation for their findings in the discussion (Line 351).

The discussion section was revised deeply. Possible explanations were given for the findings.

Line 280: “The overall treatment success rate was 234 (67.6%). The cure and treatment completion rates were 177 (51.2%) and 57 (16.5%), respectively”. How could treatment success be higher than cure rate? How was cure discriminated from treatment completion?

According to the World Health Organization, 2025 treatment outcome definition, treatment success is the sum of all patients cured and treatment completed.

Cured: a confirmed pulmonary TB case at the time of treatment initiation, who completed the treatment recommended by the national guideline, with evidence of bacteriological response and without evidence of failure.

Treatment completed: a patient who completed treatment recommended by the national policy, but the outcome does not fulfill the criteria of cure or treatment failure.

Line 294: Factors associated with treatment outcomes. HIV were associated with unfavourable treatment outcomes. If possible CD4 count and of PLWH were on ART or not would be valuable to evaluate the impact of HIV infection.

Indeed, it would have been highly valuable to include CD4 count levels and ART status of people living with HIV (PLWH) to better evaluate the impact of HIV infection on time to sputum culture conversion. However, during the piloting of our data collection tool, we found that CD4 count records for PLWH were incomplete in the study area. Therefore, we were unable to include this variable in our analysis.

6. PLOS authors have the option to publish the peer review history of their article ([what does this mean?](https://track.editorialmanager.com/CL0/https:%2F%2Fjournals.plos.org%2Fplosone%2Fs%2Feditorial-and-peer-review-process%23loc-peer-review-history/1/010f019950a6493b-af0d59ad-c452-41ff-8adb-d387bbde7e11-000000/QcrkRS_jv-rZhjdceJWPL6yp88AvZs7vmP4Fu4Xz4dY=228)). If published, this will include your full peer review and any attached files.


If you choose “no”, your identity will remain anonymous but your review may still be made public.


**Do you want your identity to be public for this peer review?** For information about this choice, including consent withdrawal, please see our [Privacy Policy](https://track.editorialmanager.com/CL0/https:%2F%2Fwww.plos.org%2Fprivacy-policy/1/010f019950a6493b-af0d59ad-c452-41ff-8adb-d387bbde7e11-000000/QGRZSjdilFV8wSmjWa2xgIPEBHjcI6_EFJcGR-sW31c=228).

Reviewer #1: **Yes:** João Paulo Cola

Reviewer #2: No

[NOTE: If reviewer comments were submitted as an attachment file, they will be attached to this email and accessible via the submission site. Please log into your account, locate the manuscript record, and check for the action link "View Attachments". If this link does not appear, there are no attachment files.]

While revising your submission, please upload your figure files to the Preflight Analysis and Conversion Engine (PACE) digital diagnostic tool, [https://pacev2.apexcovantage.com/](https://track.editorialmanager.com/CL0/https:%2F%2Fpacev2.apexcovantage.com%2F/1/010f019950a6493b-af0d59ad-c452-41ff-8adb-d387bbde7e11-000000/Zt9L5TJN2tOwgr0jZkOrV9aSS4JpTzVoDm8IY2tmlrQ=228). PACE helps ensure that figures meet PLOS requirements. To use PACE, you must first register as a user. Registration is free. Then, login and navigate to the UPLOAD tab, where you will find detailed instructions on how to use the tool. If you encounter any issues or have any questions when using PACE, please email PLOS at [figures@plos.org](mailto:figures@plos.org). Please note that Supporting Information files do not need this step.
