# Supplementary material for: Time to sputum culture conversion, treatment outcomes, and associated factors among rifampicin-resistant or multidrug-resistant tuberculosis patients in the Sidama region, Ethiopia: A retrospective follow-up study
Source: PLoS One. 2026 Apr 13;21(4):e0327705. doi: 10.1371/journal.pone.0327705 (PMC13075698; doi:10.1371/journal.pone.0327705)
Supplement: Attachment — Submitted filename: Review_Plos One-SCC_080925_second review_010226.docx [file pone.0327705.s003.docx]

**Reviewer #2 Second review 01.02.2026**

**PLOS ONE**

**Time-to-sputum culture conversion, treatment outcomes, and associated factors among multidrug-resistant tuberculosis patients in the Sidama region, Ethiopia: A retrospective follow-up study**

The authors have conducted a retrospective follow-up study on 346 patients aimed to determine the time-to-culture conversion, treatment outcomes, and associated factors among multidrug-resistant tuberculosis in the Sidama Region, Ethiopia from 2013-2024.

The authors conclude that most of the patients (87.3%) achieved culture conversion within three months. The patients with a history of retreatment faced worse outcome, pointing to serious treatment follow-up to the patients.

Such studies are very important to evaluate the treatment and factors associated with failure to improve outcomes of patients with MDR-TB. Still, the manuscript seems preliminary and has several limitations and should be substantially revised to make their data more accessible for the reader.

The Introduction is partly repetitive on the importance of the study. Still, there is no background or references on what is already known and previously published about time-to-culture conversion, treatment outcomes, and associated factors (only in Discussion). Also, the 6 months BPaLm regimen is not mentioned (**Line 72**: *“MDR-TB treatment takes longer, 18-20 months, and it is very costly”).*

A couple of previous studies are now presented as requested, but the data is given in too much detail, and it would be more effective to extract and highlight the key findings instead. The introduction is still quite long and would benefit from a more concise and streamlined form.

*(4. Yihunie Akalu T et al. Time to sputum culture conversion and its determinants among Multidrug-resistant Tuberculosis patients at public hospitals of the Amhara Regional State: A multicenter retrospective follow up study. PLoS ONE 2018. 13(6):e0199320.*

*5. Li, Qingchun et al. Time to sputum culture conversion and its predictors among patients with multidrug-resistant tuberculosis in Hangzhou, China: A retrospective cohort study. MedicineBaltimore). 2020Dec11; 99(50):e23649.*

In the Methods the definitions of various parameters should be better explained. Ex. **Line 171**: short-term, long-term and standardized treatment should be explained since this could have relevance for side effects and outcome.

The authors have now included explanations and definitions of parameters as suggested. But here they refer to “national guidelines” and “national policy” without giving references that should have been included. Also, since there are only 17 patients with MDR-TB in Table 1, it is important to know how many received Long-term regimens vs individualized treatment vs short-term regimens, vs BPaLM.

I am not qualified to evaluate all the statistical methods used but the data collection and statistical method are not very well explained.

It is not clear to me if the authors only have re-run all their statistics or also have performed new statistical analyses. Either way, they get partly different results with opposite conclusions than what they presented in the first version of the manuscript. I am not familiar with many of their statistical analyses and cannot evaluate the validity but question that the data vary.

Also, the implications of the sample size calculations are not clear as the authors did not use these data as the numbers of patients available were much fewer.

The authors have excluded the part concerning power calculations as that was not relevant since the available number of patients was much lower. Therefore, a separate “Sample size decision” section does not seem relevant and information about numbers of patients included could have been stated in the Inclusion section above. In the Stat section, the authors refer to “they” performing the analysis which seems odd as one must believe that at least some of the authors did this.

The discussion is partly repetitive of results and just state where their findings were similar or different from other studies without discussing or giving possible explanations for their findings*.* The Conclusion is also a repetition from Results and Discussion and Limitations were deficient.

The Discussion is improved but still needs substantial improvement in editing and wording and is quite repetitive and still include too many results. They consequently refer to the “the previous study” although this seems to be many earlier reports (ref 4, 24, 26, 29 etc) (*ex. 24. Weldemhret Let al. Time to Sputum Culture Conversion and Its Predictors Among Multidrug resistant Tuberculosis Patients in Tigray, Northern Ethiopia: Retrospective Cohort Study. Infection and Drug Resistance. 2023 Dec 31:3671-81.). It would have been interesting to hear the authors possible explanations for the partly diverging finding from various parts of Ethiopia as most of the previous reports were from here.*

Also, the limitation of only 13-17 cases of MDR-TB in the cohort is not discussed and how RR and MDRTB probably vary in time to conversion. Thus, the title “Time-to-sputum culture conversion, treatment outcomes, and associated factors among **multidrug-resistant tuberculosis patients….”** as misleading as most of the data presented are from RR patients.

Also, in the Result section many paragraphs are only explaining the technical statistical findings without translating to what this means in clinical practise.

The manuscript also needs substantial update and general editing concerning language, fonts (Tables), typing errors including correct and not repetitive use of abrev. as ex. Tuberculosis (TB) and Multidrug-resistant/ Rifampicin mono-resistant- TB (MDR/TB) that have been used several times.

The manuscript is improved, but still the wording and spelling require some improvements, and the use of abbreviations is still not applied consistently throughout the paper, ex multidrug-resistant tuberculosis (MDR-TB).

**Line 34** needs ref. The WHO 2024 report will most likely provide data for 2023 including for Ethiopia.

**Line 102**: such a detailed description of the country is probably not needed.

This section is still very long giving background data not all relevant for the objective of the paper.

**Line 121**: It is not clear concerning the time period when the patient data were collected: “ *The patients were registered from January 1, 2013, to June 30, 2024, from the Sidama region of Ethiopia at the Yirgalem treatment initiation center. The researchers accessed the patient data anonymously, using their medical and multidrug-resistant TB record number, from April 1 to December 31, 2024*”.

This now seems clear.

**Line 134**: “…. patients with culture-negative results at baseline, and patients diagnosed with MDR/RR-TB who did not begin anti-TB treatment..” An explanation on who these patients are and why not treated would be interesting.

The explanation is acceptable in the comments to the review.

**Line 142**: not clear if the same calculations were performed for all outcomes? Also, since the largest sample size for a single-arm cohort study was = 1977, but the total eligible RR/MDR-TB population in the region during the study period was finally 346, are the study powered to answer the research questions and are the sample size calculations of any value?

The authors have excluded the part concerning power calculations as that was not relevant since the available number of patients was much lower.

**Line 205:** Participant agreement is already stated in the section above.

**Line 209**: Write more precise concerning which patients included “*A total of 420 patients with Multidrug-resistant tuberculosis enrolled over an 11-year follow-up*”. This statement disagrees with Table 3 were also RR and INH resistance are included.

**Line 262**: *People who registered with a drug-resistance type of MDR-TB were associated with early time to-sputum culture conversion with aHR 4.7 (1-21), P = 0.041 compared with the patients with a resistance type of RR-TB, while a treatment regimen of long-term MDR-TB treatment was significantly associated with an aHR of 10.1 (2.2-45), P = 0.003 compared with the short-term treatment.* This statement is not so clear formulated and not as expected that MDR-TB should do better than rifampicin mono resistance? Still, the authors just state this and provides no possible explanation for their findings in the discussion (Line 351).

**The authors present diverging data from their previous analyses in their revised manuscript.**

Line 329: “There was no significant association between study participants' age category, sex, HIV status, resistance type, and MDR-TB treatment regimen and the culture conversion (Table3)”

As shown in Table 1 only 5% (N=17) were MDR-TB compared to 95% RR. If these 17 are further sub grouped into short-term and long-term (or even BPalM?) groups, there would be even smaller groups weakening the conclusions for MDRTB based on statistical analyses. Also, when evaluating treatment outcome only 13 MDRTB patients were included. Thus, to present these data consequently throughout the paper as obtained from MDRTB patients is questionable. Also, 270 patients (78%) received long-term regimens despite most having only rifampicin resistance which seems somewhat strange.

**Line 280**: “*The overall treatment success rate was 234 (67.6%). The cure and treatment completion rates were 177 (51.2%) and 57 (16.5%), respectively*”. How could treatment success be higher than cure rate? How was cure discriminated from treatment completion?

The authors have explained this satisfactory

**Line 294**: Factors associated with treatment outcomes. HIV were associated with unfavourable treatment outcomes. If possible CD4 count and of PLWH were on ART or not would be valuable to evaluate the impact of HIV infection.

The authors explain that these parameters were not available.
