# Supplementary material for: Time to sputum culture conversion, treatment outcomes, and associated factors among rifampicin-resistant or multidrug-resistant tuberculosis patients in the Sidama region, Ethiopia: A retrospective follow-up study
Source: PLoS One. 2026 Apr 13;21(4):e0327705. doi: 10.1371/journal.pone.0327705 (PMC13075698; doi:10.1371/journal.pone.0327705)
Supplement: Attachment — Submitted filename: Response to Reviewers second review March 2026.docx [file pone.0327705.s004.docx]

**To**
The Editor and Reviewers
PLOS ONE

**Date:** March 4, 2026

**Dear Editor,**

**Subject:** Response to Reviewers’ Comments for Manuscript ID: PONE-D-25-32357

We thank you and both reviewers for the careful evaluation of our manuscript and for the constructive comments provided. We have revised the manuscript accordingly and believe that these changes have substantially improved its clarity, rigor, and alignment between the study objectives and the data presented.

**Response to Reviewer #1**

We acknowledge the comments regarding the absence of a response letter and the minor editorial issues noted in the original submission. In the revised version, we have included a comprehensive response letter addressing all reviewer comments. In addition, we thoroughly edited the manuscript to standardize decimal places throughout the Results section, particularly for relative frequencies. We also corrected the ordering, numbering, and labeling of all figures and ensured that each figure is appropriately cited and referenced in the text.

**Response to Reviewer #2**
We appreciate the reviewer’s detailed feedback regarding the scope, content, and interpretation of the data, which prompted substantial revisions to the manuscript. First, we revised the title and related sections to better reflect the composition of the study cohort and the data presented. We now explicitly acknowledge the imbalance between MDR-TB and RR-TB patients and clearly discuss this issue as a limitation.

Second, we confirm that the dataset used in the revised manuscript is identical to that of the original submission. In response to the reviewer’s suggestions, we conducted new statistical analyses using additional and more appropriate models. These revised models yielded different effect estimates for some variables, reflecting improvements in the suitability of the statistical methods rather than changes in the underlying data. Consequently, we updated the Results, Discussion, and Limitations sections to ensure consistency with the revised analyses and to provide a clearer interpretation of the findings in light of these methodological improvements.

For ease of review, we provide a detailed, point-by-point response outlining how each concern has been addressed below.

We greatly appreciate the time and effort devoted to reviewing our work and the constructive engagement throughout the process.

Sincerely,

Wolde Abreham Geda
On behalf of all authors

**Reviewers' comments:

Reviewer's Responses to Questions**

**Comments to the Author**

1. If the authors have adequately addressed your comments raised in a previous round of review and you feel that this manuscript is now acceptable for publication, you may indicate that here to bypass the “Comments to the Author” section, enter your conflict of interest statement in the “Confidential to Editor” section, and submit your "Accept" recommendation.

Reviewer #1: All comments have been addressed

Reviewer #2: (No Response)

Thank you for your valuable comments. We have addressed all of the second reviewer’s remaining concerns line by line in our revised response.

2. Is the manuscript technically sounds, and do the data support the conclusions?

The manuscript must describe a technically sound piece of scientific research with data that supports the conclusions. Experiments must have been conducted rigorously, with appropriate controls, replication, and sample sizes. The conclusions must be drawn appropriately based on the data presented.

Reviewer #1: Yes

Reviewer #2: No

Thank you again for your comments. We have thoroughly revised the manuscript to improve its technical soundness, and the data now fully support the conclusions.

3. Has the statistical analysis been performed appropriately and rigorously?

Reviewer #1: Yes

Reviewer #2: I Don't Know

Thank you. A rigorous statistical analysis has already been completed.

4. Have the authors made all data underlying the findings in their manuscript fully available?

The [PLOS Data policy](http://track.editorialmanager.com/CL0/http:%2F%2Fwww.plosone.org%2Fstatic%2Fpolicies.action%23sharing/1/010f019c22873018-e3955a46-eddb-4e6a-9cd1-02f3a6a237d4-000000/QMu7Dw5wJYowr8fGpBK6iNXEv5v9KSxyezF4hQr3IF8=248) requires authors to make all data underlying the findings described in their manuscript fully available without restriction, with rare exception (please refer to the Data Availability Statement in the manuscript PDF file). The data should be provided as part of the manuscript or its supporting information, or deposited to a public repository. For example, in addition to summary statistics, the data points behind means, medians and variance measures should be available. If there are restrictions on publicly sharing data—e.g. participant privacy or use of data from a third party—those must be specified.

Reviewer #1: Yes

Reviewer #2: Yes

Thank you.

5. Is the manuscript presented in an intelligible fashion and written in Standard English?

PLOS ONE does not copyedit accepted manuscripts, so the language in submitted articles must be clear, correct, and unambiguous. Any typographical or grammatical errors should be corrected at revision, so please note any specific errors here.

Reviewer #1: Yes

Reviewer #2: No

Thank you for your comments. Our detailed responses to Reviewer #2’s concerns are provided below in the section “Responses to Reviewer #2 (Second Review, 01.02.202x).”

6. Review Comments to the Author

Please use the space provided to explain your answers to the questions above. You may also include additional comments for the author, including concerns about dual publication, research ethics, or publication ethics. (Please upload your review as an attachment if it exceeds 20,000 characters)

**Reviewer #1:** The authors implemented the revisions addressing the points raised in the review; however, they did not submit a response letter. The manuscript still requires minor editing to standardize decimal places in the results (particularly for relative frequencies) and to correct the ordering/numbering of the figures, as some figures are not cited in the text and/or are mislabeled.

**Reviewer #2:** The authors have improved the paper but are still needs substantial revision in content and editing. The main objection is that the authors aim to present data from MDR-TB patients whereas only 5% or less of the cohort are MDR-TB, Thus, the title “Time-to-sputum culture conversion, treatment outcomes, and associated factors among multidrug-resistant tuberculosis patients….” as misleading as most of the data presented are from RR patients and this is not discussed as a limitation. Also, they present data partly diverging from the first version and it is unclear if this is a result of re-analyses correcting previous results or new statistical methods.

We thank both reviewers for your careful evaluation of our manuscript and for the constructive comments provided. We have revised the manuscript accordingly and believe that these changes have substantially improved its clarity, rigor, and alignment between the study objectives and the data presented.

**Response to Reviewer #1**

We acknowledge the reviewer’s comments regarding the absence of a response letter and the noted minor editorial issues. In the revised submission, we have included a comprehensive response letter addressing all reviewer comments.

In addition, we have thoroughly edited the manuscript to standardize decimal places throughout the Results section, particularly for relative frequencies. We have also corrected the ordering, numbering, and labeling of all figures and ensured that each figure is appropriately cited and referenced in the text.

**Response to Reviewer #2**

We appreciate the reviewer’s detailed feedback regarding the scope, content, and interpretation of the data. In response to these concerns, we have undertaken substantial revisions to the manuscript.

First, we revised the title and related sections to better reflect the composition of the study cohort and the data presented. We now explicitly acknowledge the imbalance between MDR-TB and RR-TB patients and have clearly discussed this issue as a limitation in the Limitations section.

Second, we confirm that the dataset used in the revised manuscript is identical to that of the original submission. In response to the reviewer’s suggestions, we conducted new statistical analyses using additional and more appropriate models. These revised models resulted in different effect estimates for some variables. These differences reflect improvements in the suitability of the statistical methods rather than changes in the underlying data.

Finally, we have updated the Results, Discussion, and limitation sections to ensure consistency with the revised analyses and to provide a clearer interpretation of the findings in light of these methodological improvements.

7. PLOS authors have the option to publish the peer review history of their article ([what does this mean?](https://track.editorialmanager.com/CL0/https:%2F%2Fjournals.plos.org%2Fplosone%2Fs%2Feditorial-and-peer-review-process%23loc-peer-review-history/1/010f019c22873018-e3955a46-eddb-4e6a-9cd1-02f3a6a237d4-000000/B6x4Y1j3MyVqKdMfmiue_rStGCkFopG_f68aJOLX5bY=248)). If published, this will include your full peer review and any attached files.


If you choose “no”, your identity will remain anonymous but your review may still be made public.


**Do you want your identity to be public for this peer review?** For information about this choice, including consent withdrawal, please see our [Privacy Policy](https://track.editorialmanager.com/CL0/https:%2F%2Fwww.plos.org%2Fprivacy-policy/1/010f019c22873018-e3955a46-eddb-4e6a-9cd1-02f3a6a237d4-000000/3JmVIlyIKg8_bGEr7MV7takqZBm7HrZ3-_ZsP7nzKRY=248).

Reviewer #1: **Yes:** João Paulo Cola

Reviewer #2: **Yes:** Anne Ma Dyrhol-Riise

[NOTE: If reviewer comments were submitted as an attachment file, they will be attached to this email and accessible via the submission site. Please log into your account, locate the manuscript record, and check for the action link "View Attachments". If this link does not appear, there are no attachment files.]

To ensure your figures meet our technical requirements, please review our figure guidelines: https://journals.plos.org/plosone/s/figures

You may also use PLOS’s free figure tool, NAAS, to help you prepare publication quality figures: https://journals.plos.org/plosone/s/figures#loc-tools-for-figure-preparation.

NAAS will assess whether your figures meet our technical requirements by comparing each figure against our figure specifications.

*In compliance with data protection regulations, you may request that we remove your personal registration details at any time.* [*(Remove my information/details)*](https://track.editorialmanager.com/CL0/https:%2F%2Fwww.editorialmanager.com%2Fpone%2Flogin.asp%3Fa=r/1/010f019c22873018-e3955a46-eddb-4e6a-9cd1-02f3a6a237d4-000000/B9rLoN7tpu9oHd3Siy32ub8833A6aniSaY8oTVV7heY=248)*. Please contact the publication office if you have any questions.*

**Responses to Reviewer #2 second review 01.02.2026**

**PLOS ONE**

**Time-to-sputum culture conversion, treatment outcomes, and associated factors among multidrug-resistant tuberculosis patients in the Sidama region, Ethiopia: A retrospective follow-up study**

The authors have conducted a retrospective follow-up study on 346 patients aimed to determine the time-to-culture conversion, treatment outcomes, and associated factors among multidrug-resistant tuberculosis in the Sidama Region, Ethiopia from 2013-2024.

The authors conclude that most of the patients (87.3%) achieved culture conversion within three months. The patients with a history of retreatment faced worse outcome, pointing to serious treatment follow-up to the patients.

Such studies are very important to evaluate the treatment and factors associated with failure to improve outcomes of patients with MDR-TB. Still, the manuscript seems preliminary and has several limitations and should be substantially revised to make their data more accessible for the reader.

The Introduction is partly repetitive on the importance of the study. Still, there is no background or references on what is already known and previously published about time-to-culture conversion, treatment outcomes, and associated factors (only in Discussion). Also, the 6 months BPaLm regimen is not mentioned (**Line 72**: *“MDR-TB treatment takes longer, 18-20 months, and it is very costly”).*

A couple of previous studies are now presented as requested, but the data is given in too much detail, and it would be more effective to extract and highlight the key findings instead. The introduction is still quite long and would benefit from a more concise and streamlined form.

*(4. Yihunie Akalu T et al. Time to sputum culture conversion and its determinants among Multidrug-resistant Tuberculosis patients at public hospitals of the Amhara Regional State: A multicenter retrospective follow up study. PLoS ONE 2018. 13(6):e0199320.*

*5. Li, Qingchun et al. Time to sputum culture conversion and its predictors among patients with multidrug-resistant tuberculosis in Hangzhou, China: A retrospective cohort study. MedicineBaltimore). 2020Dec11; 99(50):e23649.*

Thank you for your insightful comments. In response, we have revised the Introduction to enhance clarity and conciseness. The descriptions of previous studies by Yihunie Akalu T et al. and Li et al. have been substantially streamlined by removing excessive methodological and numerical details. Only the key findings relevant to time to sputum culture conversion and its main determinants are now highlighted. As a result, the Introduction has been shortened and reorganized to present the background in a more focused and effective manner.

In the Methods the definitions of various parameters should be better explained. Ex. **Line 171**: short-term, long-term and standardized treatment should be explained since this could have relevance for side effects and outcome.

The authors have now included explanations and definitions of parameters as suggested. But here they refer to “national guidelines” and “national policy” without giving references that should have been included. Also, since there are only 17 patients with MDR-TB in Table 1, it is important to know how many received Long-term regimens vs individualized treatment vs short-term regimens, vs BPaLM.

We thank the Reviewer for this important observation. We have referenced WHO consolidated guidelines on tuberculosis. Module 4: treatment - drug-resistant tuberculosis treatment. Geneva: World Health Organization; 2020. Licence: CC BY-NC-SA 3.0 IGO (Ref. 8), because it contains well-articulated definitions in regard to the TB and MDR-TB treatments.

Regarding the limited number of patients with MDR-TB (n = 17) presented in Table 1, we agree that further disaggregation of treatment regimens (long-term standardized, individualized, short-term, and BPaLM) would ideally provide additional clinical insights. We explored this approach during the initial analysis; however, several regimen categories contained tiny numbers of patients, precluding meaningful statistical analysis and reliable interpretation.

To address this limitation while preserving analytical robustness, we therefore grouped treatment regimens into two widely accepted categories:
(i) **Long-term treatment regimens**, including both standardized and individualized approaches; and
(ii) **Short-term treatment regimens**, comprising 9–12-month regimens and the 6-month BPaLM regimen.

This classification aligns with current programmatic approaches and allows for more stable comparisons within the constraints of the available data.

I am not qualified to evaluate all the statistical methods used but the data collection and statistical method are not very well explained.

It is not clear to me if the authors only have re-run all their statistics or also have performed new statistical analyses. Either way, they get partly different results with opposite conclusions than what they presented in the first version of the manuscript. I am not familiar with many of their statistical analyses and cannot evaluate the validity but question that the data vary.

Thank you for this valuable comment. The dataset used in the revised manuscript is identical to that used in the original submission. In response to the reviewer’s suggestions, we performed new statistical analyses using additional and more appropriate models, rather than simply re-running the original analyses.

These revised models led to different estimates for some effects and, consequently, to revised inferences compared with the original version of the manuscript. The differences in conclusions therefore arise from the improved suitability of the statistical models, not from changes or variability in the data.

We have updated the Results, Discussion, and Conclusions sections accordingly to ensure that they accurately reflect the revised analyses and their interpretation.

Also, the implications of the sample size calculations are not clear as the authors did not use these data as the numbers of patients available were much fewer.

The authors have excluded the part concerning power calculations as that was not relevant since the available number of patients was much lower. Therefore, a separate “Sample size decision” section does not seem relevant and information about numbers of patients included could have been stated in the Inclusion section above. In the Stat section, the authors refer to “they” performing the analysis which seems odd as one must believe that at least some of the authors did this.

We sincerely thank the reviewer for the constructive comments. The indicated sections have been revised in accordance with the suggestions, and the sample size decision was merged to the inclusion section. Additionally, the personal pronoun “they” has been changed to “we,” as recommended.

The discussion is partly repetitive of results and just state where their findings were similar or different from other studies without discussing or giving possible explanations for their findings*.* The Conclusion is also a repetition from Results and Discussion and Limitations were deficient.

The Discussion is improved but still needs substantial improvement in editing and wording and is quite repetitive and still include too many results. They consequently refer to the “the previous study” although this seems to be many earlier reports (ref 4, 24, 26, 29 etc) (*ex. 24. Weldemhret Let al. Time to Sputum Culture Conversion and Its Predictors Among Multidrug resistant Tuberculosis Patients in Tigray, Northern Ethiopia: Retrospective Cohort Study. Infection and Drug Resistance. 2023 Dec 31:3671-81.). It would have been interesting to hear the authors possible explanations for the partly diverging finding from various parts of Ethiopia as most of the previous reports were from here.*

Also, the limitation of only 13-17 cases of MDR-TB in the cohort is not discussed and how RR and MDRTB probably vary in time to conversion. Thus, the title “Time-to-sputum culture conversion, treatment outcomes, and associated factors among **multidrug-resistant tuberculosis patients….”** as misleading as most of the data presented are from RR patients.

Also, in the Result section many paragraphs are only explaining the technical statistical findings without translating to what this means in clinical practise.

We sincerely thank the reviewer for these detailed and constructive comments. In response, we have undertaken substantial revisions to the manuscript to improve clarity, coherence, and scientific rigor.

**1. Revision of the Discussion section**
The Discussion has been extensively edited to reduce repetition, improve wording, and minimize the presentation of detailed results. We now focus on synthesizing the major findings and their implications.

**2. Diverging findings across regions in Ethiopia**
As suggested, we have added a new paragraph discussing possible explanations for the partly diverging findings reported across different regions of Ethiopia. These include variations in patient characteristics, programmatic implementation of MDR/RR-TB treatment, diagnostic capacity, adherence support, and timing of culture monitoring.

**3. Limited number of MDR-TB cases and distinction from RR-TB**

We now explicitly address the limitation related to the relatively small number of MDR-TB cases (13–17 patients) compared with RR-TB cases. This has been discussed in the Limitations section, including its potential impact on statistical power and interpretation of subgroup analyses. We also discussed that, in our cohort, drug resistance type (MDR-TB versus RR-TB) was not significantly associated with time to sputum culture conversion.

**4. Title and use of the term “multidrug-resistant tuberculosis”**
We appreciate the reviewer’s important comment regarding the potential for a misleading title, given that most participants had rifampicin-resistant TB (RR-TB). While RR-TB constituted the majority of cases, we initially used the term “multidrug-resistant tuberculosis” because:

(i) In routine clinical and programmatic practice, RR-TB is often managed as MDR-TB, as a high proportion (approximately 80–90%) of RR-TB cases are also resistant to isoniazid, as documented in prior studies;
(ii) MDR-TB and RR-TB patients typically receive similar second-line treatment regimens; and
(iii) This terminology is commonly used in national and international guidelines.

To address the reviewer’s concern and improve clarity, we have revised the title to:
“Time-to-sputum culture conversion, treatment outcomes, and associated factors among multidrug-resistant or rifampicin-resistant tuberculosis patients in the Sidama region, Ethiopia: A retrospective follow-up study.”

**5. Clinical interpretation of statistical findings**

We have revised the Results section to better translate statistical findings into clinically meaningful interpretations.

The manuscript also needs substantial update and general editing concerning language, fonts (Tables), typing errors including correct and not repetitive use of abrev. as ex. Tuberculosis (TB) and Multidrug-resistant/ Rifampicin mono-resistant- TB (MDR/TB) that have been used several times.

The manuscript is improved, but still the wording and spelling require some improvements, and the use of abbreviations is still not applied consistently throughout the paper, ex multidrug-resistant tuberculosis (MDR-TB).

According to your comments we have made substantial editing, revising wordings, correcting spellings and standardizing abbreviations.

**Line 34** needs ref. The WHO 2024 report will most likely provide data for 2023 including for Ethiopia.

**Line 102**: such a detailed description of the country is probably not needed.

This section is still very long giving background data not all relevant for the objective of the paper.

**Line 121**: It is not clear concerning the time period when the patient data were collected: “ *The patients were registered from January 1, 2013, to June 30, 2024, from the Sidama region of Ethiopia at the Yirgalem treatment initiation center. The researchers accessed the patient data anonymously, using their medical and multidrug-resistant TB record number, from April 1 to December 31, 2024*”.

This now seems clear.

**Line 134**: “…. patients with culture-negative results at baseline, and patients diagnosed with MDR/RR-TB who did not begin anti-TB treatment..” An explanation on who these patients are and why not treated would be interesting.

The explanation is acceptable in the comments to the review.

**Line 142**: not clear if the same calculations were performed for all outcomes? Also, since the largest sample size for a single-arm cohort study was = 1977, but the total eligible RR/MDR-TB population in the region during the study period was finally 346, are the study powered to answer the research questions and are the sample size calculations of any value?

The authors have excluded the part concerning power calculations as that was not relevant since the available number of patients was much lower.

**Line 205:** Participant agreement is already stated in the section above.

**Line 209**: Write more precise concerning which patients included “*A total of 420 patients with Multidrug-resistant tuberculosis enrolled over an 11-year follow-up*”. This statement disagrees with Table 3 were also RR and INH resistance are included.

**Line 262**: *People who registered with a drug-resistance type of MDR-TB were associated with early time to-sputum culture conversion with aHR 4.7 (1-21), P = 0.041 compared with the patients with a resistance type of RR-TB, while a treatment regimen of long-term MDR-TB treatment was significantly associated with an aHR of 10.1 (2.2-45), P = 0.003 compared with the short-term treatment.* This statement is not so clear formulated and not as expected that MDR-TB should do better than rifampicin mono resistance? Still, the authors just state this and provides no possible explanation for their findings in the discussion (Line 351).

**The authors present diverging data from their previous analyses in their revised manuscript.**

Line 329: “There was no significant association between study participants' age category, sex, HIV status, resistance type, and MDR-TB treatment regimen and the culture conversion (Table3)”

As shown in Table 1 only 5% (N=17) were MDR-TB compared to 95% RR. If these 17 are further sub grouped into short-term and long-term (or even BPalM?) groups, there would be even smaller groups weakening the conclusions for MDRTB based on statistical analyses. Also, when evaluating treatment outcome only 13 MDRTB patients were included. Thus, to present these data consequently throughout the paper as obtained from MDRTB patients is questionable. Also, 270 patients (78%) received long-term regimens despite most having only rifampicin resistance which seems somewhat strange.

Thank you for these insightful comments. We would like to clarify that the revised manuscript does not present a divergent dataset. In response to earlier reviewer feedback, we collapsed previously extended subgroups into fewer, conceptually related categories to increase the number of observations per group; however, the underlying dataset remained unchanged.

As part of the revision, we also re-ran the analyses using additional and more appropriate statistical models. The observed differences compared with prior analyses reflect improved model specification and calibration rather than inconsistencies in the data.

We acknowledge the small number of MDR-TB patients (n = 17), and the further reduction when stratified by treatment regimen or treatment outcome (n = 13). This limited sample size restricts the statistical power and strength of MDR-TB–specific inferences. Accordingly, we have avoided overinterpretation of MDR-TB–specific findings and have explicitly highlighted this limitation in the revised manuscript.

Regarding the apparent imbalance in treatment regimens, with 78% of patients receiving long-term regimens despite rifampicin resistance, this reflects programmatic practice during the study period. Previous studies from similar settings have reported that approximately 80–90% of rifampicin-resistant TB cases are also resistant to isoniazid, leading clinicians to preferentially prescribe long-term regimens. In addition, long-term regimens were the most widely used treatment option available during the study period.

.

**Line 280**: “*The overall treatment success rate was 234 (67.6%). The cure and treatment completion rates were 177 (51.2%) and 57 (16.5%), respectively*”. How could treatment success be higher than cure rate? How was cure discriminated from treatment completion?

The authors have explained this satisfactory

**Line 294**: Factors associated with treatment outcomes. HIV were associated with unfavourable treatment outcomes. If possible CD4 count and of PLWH were on ART or not would be valuable to evaluate the impact of HIV infection.

The authors explain that these parameters were not available.
